# A Twin-Cysteine Motif in the V2 Region of gp120 Is Associated with SIV Envelope Trimer Stabilization

**DOI:** 10.1371/journal.pone.0069406

**Published:** 2013-07-23

**Authors:** Christopher Bohl, Dane Bowder, Jesse Thompson, Levon Abrahamyan, Sandra Gonzalez-Ramirez, Youdong Mao, Joseph Sodroski, Charles Wood, Shi-hua Xiang

**Affiliations:** 1 Nebraska Center for Virology, University of Nebraska-Lincoln, Lincoln, Nebraska, United States of America; 2 School of Veterinary Medicine and Biomedical Sciences, University of Nebraska-Lincoln, Lincoln, Nebraska, United States of America; 3 School of Biological Sciences, University of Nebraska-Lincoln, Lincoln, Nebraska, United States of America; 4 Department of Cancer Immunology and AIDS, Dana-Farber Cancer Institute, Boston, Massachusetts, United States of America; 5 Department of Microbiology and Immunology, Division of AIDS, Harvard Medical School, Boston, Massachusetts, United States of America; 6 Department of Immunology and Infectious Diseases, Harvard School of Public Health, Boston, Massachusetts, United States of America; 7 Ragon Institute of Massachusetts General Hospital, Massachusetts Institute of Technology and Harvard University, Boston, Massachusetts, United States of America; University of Missouri, United States of America

## Abstract

The V1 and V2 variable regions of the primate immunodeficiency viruses contribute to the trimer association domain of the gp120 exterior envelope glycoprotein. A pair of V2 cysteine residues at 183 and 191 (“twin cysteines”) is present in several simian immunodeficiency viruses, human immunodeficiency virus type 2 (HIV-2) and some SIV_cpz_ lineages, but not in HIV-1. To examine the role of this potentially disulfide-bonded twin-cysteine motif, the cysteine residues in the SIVmac239 envelope glycoproteins were individually and pairwise substituted by alanine residues. All of the twin-cysteine mutants exhibited decreases in gp120 association with the Env trimer, membrane-fusing activity, and ability to support virus entry. Thus, the twin-cysteine motif plays a role in Env trimer stabilization in SIV and may do so in HIV-2 and some SIV_cpz_ as well. This implies that HIV-1 lost the twin-cysteines, and may have relatively unstable Env trimers compared to SIV and HIV-2.

## Introduction

It has been well established that human immunodeficiency viruses (HIV) are derived from simian immunodeficiency viruses (SIV) through cross-species transmission [1]–[4]. The introduction of primate lentiviruses (PLV) into new host species can result in pathogenesis. Unlike SIV in nonhuman primates, HIV-1 frequently causes human immune system failure and leads to fatal acquired immunodeficiency syndrome (AIDS) [5]–[7]. HIV-1 has infected more than 60 million people and caused the AIDS-related deaths of 25 million people globally (UNAIDS, Report on Globe AIDS Epidemic 2010). The basis for the differences between pathogenic HIV-1 infections in humans and the generally apathogenic SIV infections in African monkeys is not well understood. In the latter case, SIV and the host immune system apparently achieve a mutual balance. However, if the host loses the ability to control the virus, disease can result, as observed with HIV-1 in humans [8]–[11]. Of interest, SIVcpz in chimpanzees, which represents the intermediate in PLV transmission from non-human primates to humans, can also exhibit pathogenicity in chimpanzees resembling that of HIV-1 in humans [12]–[15]. Thus, SIV infection of monkeys, SIVcpz infection of chimpanzees, and HIV-1 infection of humans apparently represent examples of progressively poorer host immune system control of virus and increased pathogenicity.

The PLV, which include HIV-1, HIV-2 and SIV, are enveloped retroviruses. The trimeric envelope glycoprotein (Env) spikes on the virion surface are the only viral molecules making direct contacts with host cell receptors (CD4 and a chemokine receptor like CCR5). PLV Env evolution is driven by requirements to mediate host cell entry and to evade host-generated neutralizing antibodies. HIV and SIV Envs evade host immunity by employing mechanisms such as rapid alteration of surface loops, glycan shielding and presentation of multiple conformers, which may act as decoys [16]–[22]. An understanding of HIV/SIV Env structure has relied on X-ray crystal structures of fragments of the gp120 and gp41 subunits and on low-resolution cryo-electron microscopic reconstructions [8]–[11]. Unfortunately, the three-dimensional structure of the trimeric spike is still unknown, despite significant effort. However, cryo-electron tomography and single-particle electron microscopy approaches have yielded significant insights. Lower-resolution native Env trimer architectures on HIV-1 and SIV have been described [23]–[26]. It has been suggested that the V1V2 regions are located at the membrane-distal apex of the Env spike [23], [27]. If the V1V2 regions are deleted, the SIV Env trimer can assume a more open and flexible structure [28]. The recently reported HIV-1 Env trimer structure at 11-Å resolution shows that the V1, V2 and V3 variable regions of the gp120 exterior Env subunit interact near the trimer axis [8]–[11]. This trimer-association domain (TAD) of gp120 potentially regulates trimer stability and other HIV/SIV Env phenotypes [29].

We are particularly interested in the participation of the V2 region in the trimer association domain (TAD) because so little is known about its structure and function. However, the V2 region is immunogenic, and in some cases serves as a target for broadly cross-reactive neutralizing antibodies [11], [30]–[34]. Recent data from antibody complexes with V2 peptides indicate that the V2 region of HIV-1 can potentially assume multiple conformations, depending on context [35], [36].

**Figure 1:**
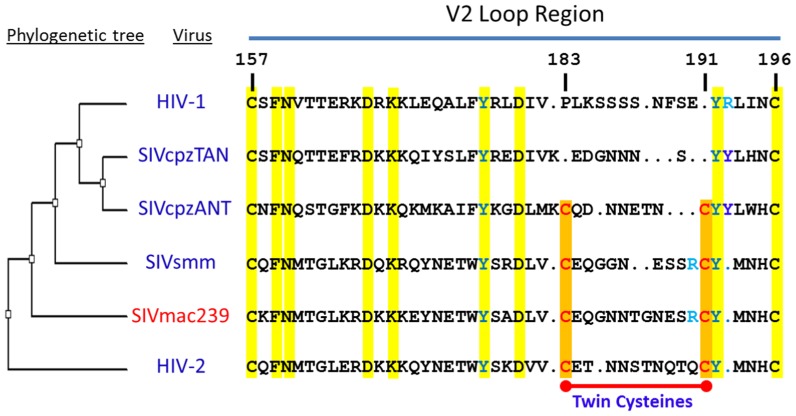
Phylogeny of the gp120 V2 region in PLVs. The Phylogenetic tree (unrooted, left panel) was constructed based on whole genome nucleotide sequences, using a neighbor-joining method [43] and bootstrapping for 1000 steps. The associated gp120 V2 region sequence alignment was accomplished using the Clustal W program [44]. The twin-cysteine residues are colored red and marked with a red bar, and the conserved tyrosine (Y) in green. The residues are numbered based on the standard system, which uses HXBc2 as a reference [45]. The viral strains used for the phylogenetic tree construction are as follows respectively: HIV-1, AF033819; HIV-2, M30502; SIVcpzANT, U42720; SIVcpzTAN, EF394356; SIVsmm, AF4679.

Alignment of the PLV V2 sequences revealed the presence of two conserved cysteine residues in HIV-2 and SIV strains (Figure 1). Because these two cysteines are always either present or absent as a pair during PLV evolution, we will refer to them as “twin cysteines”. This distinguishes them from other cysteine residues within the gp120 molecule, the disulfide-bonding pattern of which is well established [37]–[40]. The twin cysteines in the gp120 V2 region of most SIV and HIV-2 strains are absent in all HIV-1 strains. We hypothesize that these twin cysteines may form a disulfide bond that plays a role in envelope trimer stabilization. Here, we provide data supporting this hypothesis, and show that the twin cysteines of SIVmac239 contribute significantly to stabilizing the non-covalent interaction of gp120 within the Env trimer.

## Materials and Methods

### Cell Lines

The 293T cells used for SIV envelope expression and recombined viruses production were grown on Dulbecco’s modified Eagle medium (DMEM) containing 10% fetal bovine serum and 100 µg/ml of penicillin/streptomycin at 37°C and 5% CO_2_. The cell lines based on Cf2Th-CD4 cells were grown on complete DMEM with 150 µg/ml of hygromycin medium but supplemented with additional antibiotics, 50 µg/ml G418 for Cf2Th-CD4/CCR5 cells, 300 ug/ml Zeocin for CfTh2-CD4/CXCR4 cells. The TZM-bl reporter cell line (NIH AIDS Research and Reagent Program) was used for viral infection experiments as it expresses CD4 and CCR5, and also contains both beta-galactosidase and luciferase reporter genes; the TZM-bl cell line was grown in normal complete DMEM medium.

### Site-directed Mutagenesis

The twin-cysteine substitution mutants (183A/C and 191C/A, 183C/A+191C/A) as well as other related mutants (192Y/A and 190R/E) were made in the pcDNA3.1(+) vector using site-directed mutagenesis (QuikChange II XL kit from Stratagene). All mutagenesis primers were synthesized by Integrated DNA Technologies (IDT). DNA sequencing was used to verify all mutations generated by the designed primers.

### Envelope Expression, Processing and Shedding Assay

Gel electrophoresis was used to check the envelope glycoprotein expression, processing and stability, and also used to separate gp160 and gp120 Env glycoproteins. This method is based on radioactive (^35^S methionine/cysteine) metabolic labeling of the Env-expressing cells and immunoprecipitation [41]. In brief, the Env-expressing plasmid was transfected into 293T cells. One day after transfection, the cells were metabolically labeled with ^35^S-(protein-labeling mix) (Perkin-Elmer). After overnight culturing, the media and the cell lysates were harvested for analysis. The radiolabeled envelope proteins were precipitated using anti-SIV serum with Protein A-Sepharose beads, followed by SDS-PAGE gel analysis.

### Pulse-chase Labeling

The transfected 293T cells were cultured in methionine/cysteine-free medium for 30 minutes followed by pulse labeling for 1 hour with 300 uCi of [^35^S]-methionine/cysteine (>1000 Ci/mmol; NEN). The labeling medium was removed and the cells were washed with complete medium and chased in complete medium for 2, 4, and 8 hours. At the appropriate times, medium was collected, clarified by micro-centrifugation, and adjusted to 1X lysis buffer containing protease inhibitors. The cells were lysed in 1X lysis buffer containing protease inhibitors and clarified by micro-centrifugation. Lysates were pre-cleared with normal monkey serum and viral proteins were immunoprecipitated with SIVmac251-infected monkey serum, separated by SDS-PAGE, and analyzed by phosphoimaging using the Discovery Series Quantity One software (Bio-Rad).

### Ligand Binding

The wild-type or a mutant envelope plasmid was transfected into 293T cells using the Effectene transfection reagent (Qiagen). After 2 days, the transfected cells were metabolically labeled using ^35^S-methionine/cysteine (1000 Ci/mmol, Perkin-Elmer) overnight in DMEM medium free of methionine and cysteine. The medium was collected and the cells were lysed with buffer containing a lower NP-40 concentration. After centrifugation to remove the cell debris, the supernatant and the collected medium were mixed for the ligand binding experiments. The CD4 binding was carried out by using human Ig-tagged CD4 protein (CD4-IgG2 from the NIH AIDS Research and Reagent Program) with the radiolabeled viral envelope glycoproteins. The bound envelope proteins were analyzed by SDS-PAGE and exposure to X-ray film. The assessment of gp120 binding to the co-receptor CCR5 was done by using CF2Th-CCR5 cells in the presence of sCD4 (10 µg/ml). The CCR5-expressing cells were incubated with the radiolabeled gp120 envelope glycoproteins in the supernatants of Env-expressing cells. The CCR5-expressing cells were washed, lysed, and precipitated with HIV-1 positive pertinent sera and Protein-A beads. The immunoprecipitates were applied to an SDS-PAGE gel, and then visualized and quantified after exposing to X-ray film or using a phosphoimager.

### Cell Fusion Assay

The fusion abilities of all the Env mutants were measured using TZM-bl target cells that express the HIV/SIV receptors CD4 and coreceptor CCR5 and CXCR4. The 293T cells in 6-well plates were co-transfected with the Env-expressing plasmids and a Tat-expressing plasmid using Fugene6 [42] according to the manufacturer’s instructions. The transfected cells (effector cells) were incubated for 24 hours, harvested and washed, then overlaid on TZM-bl target cells seeded onto a 96-well plate to measure cell-to-cell fusion. The fusion activity between effector and donor cells can be readily measured by luciferase activity. After overnight co-culturing, the cells were washed once with PBS, lysed and luciferase activity was measured by using the Luciferase Assay System (Promega) and the Veritas luminometer (Turner Biosystems). The relative light units (RLU) representing the luminescence indicated the amount of cell-cell fusion. The background luminescence produced by co-cultured mock-transfected 293T and TMZ-bl cells was subtracted from the RLU values observed in wild type (wt) and mutant Env-transfected cells.

### Viral Infection Analysis

The viral infection analysis was carried out using TZM-bl as target cells. The wild-type and twin-cysteine or other Env mutants were pseudotyped on viruses using the viral genomic backbone plasmid pNL-4-3_GFPΔEnv in transfected 293T cells. The viral titers were measured by RT activity using liquid scintillation to detect incorporation of H^3^ into newly synthesized DNA; input virus for infections was normalized as CPM reflecting functional reverse transcriptase in virus stocks. The infectivity of the wild-type and mutant viruses was determined by incubating the recombinant virions with TZM-bl target cells for 48 hours at 37°C. The GFP intensity was measured using flow cytometry.

## Results

### Twin-cysteine SIV gp120 Mutants Exhibit Reduced Infectivity

To investigate the role of the twin cysteines during viral infection, we altered them in the prototypic SIV strain SIVmac239 and tested the effect of these changes on virus infectivity. The twin cysteines were changed singly (183C/A, 191C/A) or together (183C/A+191C/A). Additionally, we altered a conserved tyrosine (192Y) located adjacent to the second cysteine (192Y/A) (Figure 1). Finally, we altered the less conserved arginine (R) at position 190, changing this residue to glutamic acid (190R/E).

**Figure 2:**
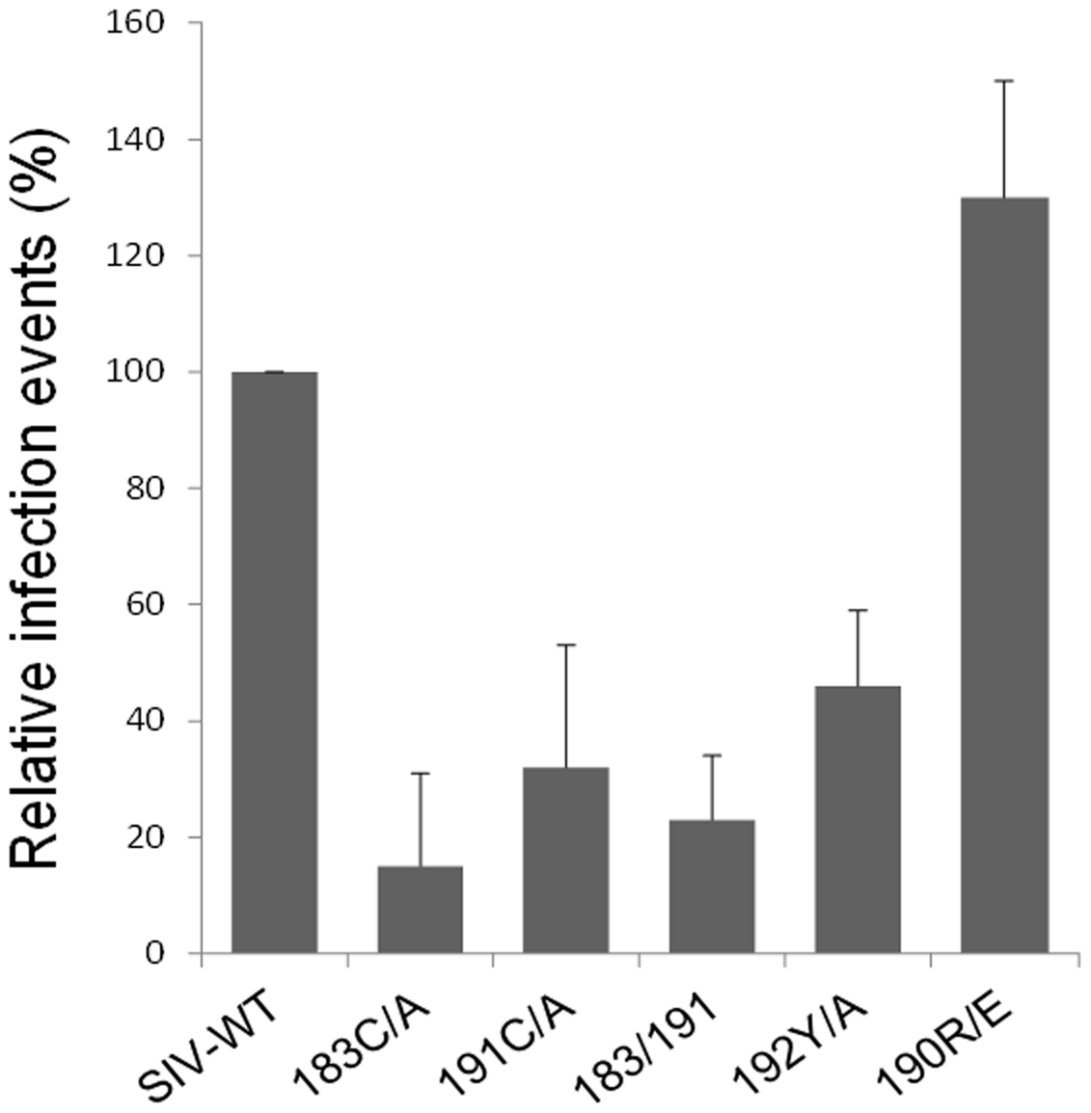
Infectivity of the twin-cysteine mutants and other gp120 V2 region mutants of SIVmac239. Viruses pseudotyped with the wild-type (wt) and mutant SIVmac239 Envs were made with a GFP-expressing vector and used to infect TZM-bl target cells. The infectivity was evaluated by measuring the fluorescence intensity of GFP by flow cytometry.

The relative infectivity of the SIVmac239 V2 mutants is shown in Figure 2. All of the twin cysteine mutants exhibited significant reductions in infectivity. This was also true for the 192Y/A mutant. However, the 190R/E mutant efficiently supported virus infection. Thus, alteration of conserved V2 residues near or within the twin-cysteine motif can significantly reduce the ability of Env to mediate SIVmac239 infection.

### Changes in the Twin Cysteines Reduce Cell-cell Fusion

**Figure 3:**
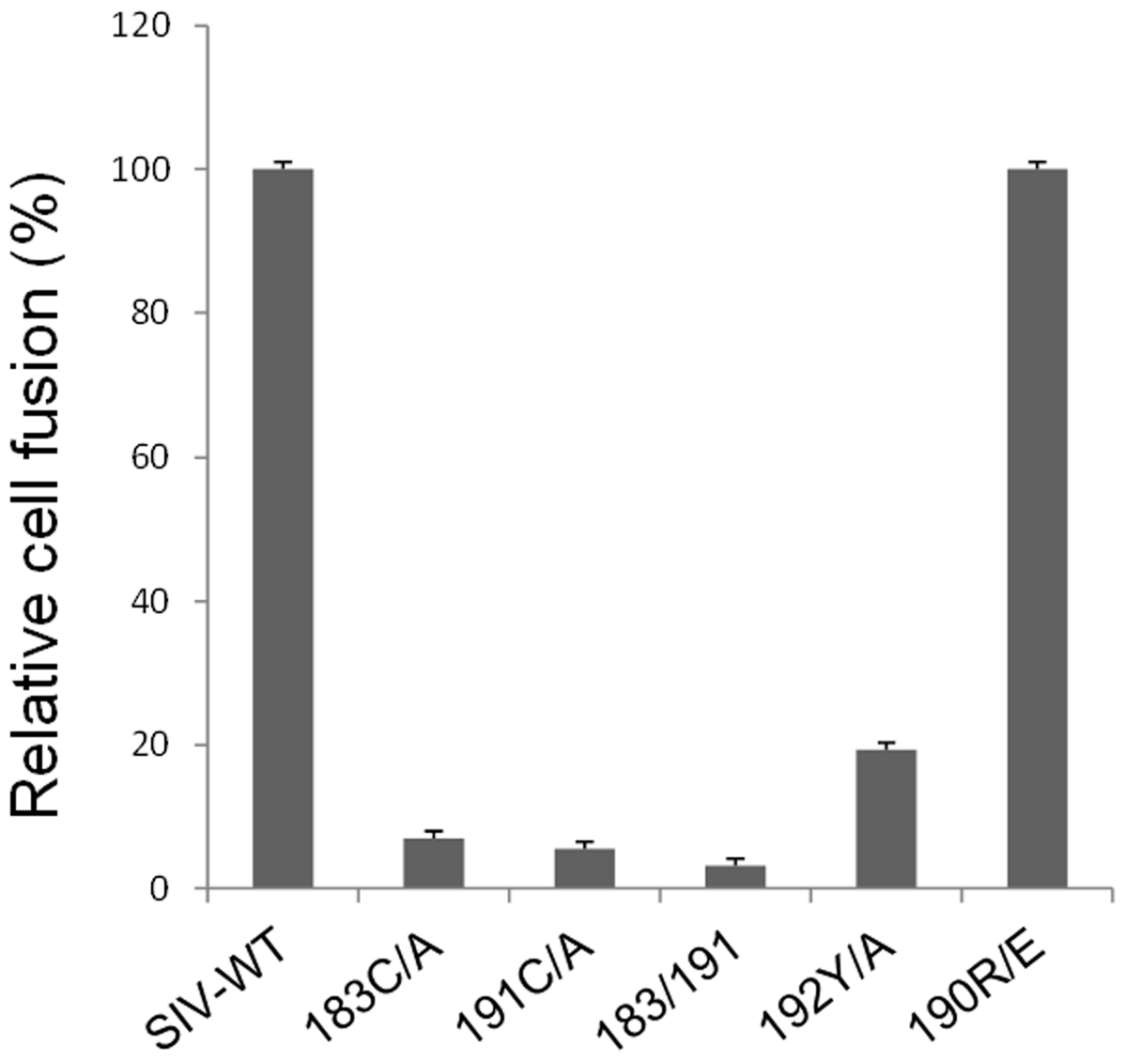
Cell-cell fusion mediated by SIVmac239 Env variants. The cell-cell fusion assay was performed by measuring the fluorescence intensity of Tat-inducible luciferase resulting from fusion of the SIVmac239 Env-expressing cells with target TZM-bl cells.

We tested the ability of the SIVmac239 mutants to mediate cell-cell fusion. The twin-cysteine mutants were significantly reduced in their cell-cell fusion ability, along with the 192Y/A mutant (Figure 3). The 190R/E mutant supported cell-cell fusion as efficiently as the wild-type SIVmac239 Env. Thus, the cell-cell fusion data correlate nicely with the single-round virus infection data, suggesting that the twin-cysteine and 192Y/A mutants exhibit decreased Env functionality in mediating membrane fusion.

### Twin-cysteine Changes do not Affect Receptor Binding

**Figure 4:**
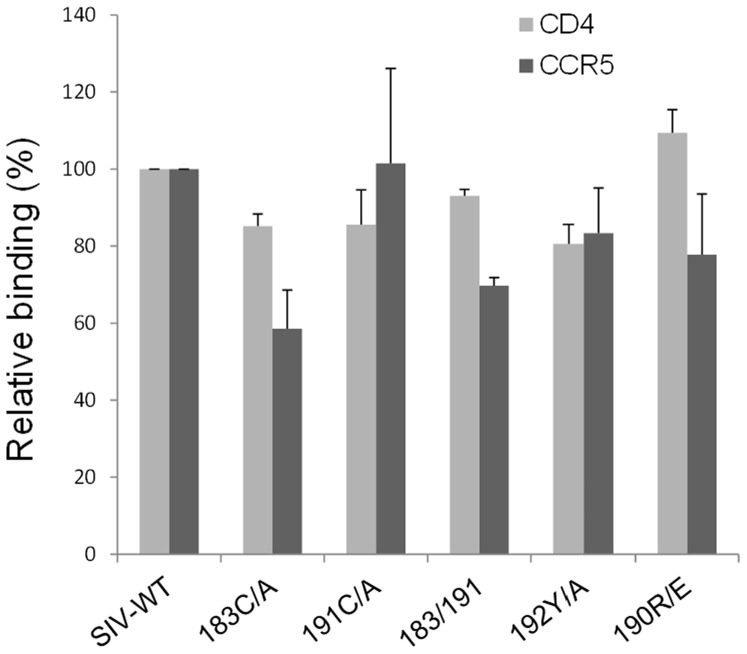
Receptor binding of SIVmac239 Env variants. The assay measuring gp120 binding to human CD4 and human CCR5 utilized ^35^S-radiolabeled gp120 precipitated with CD4-Ig or incubated with CCR5-expressing cells in the presence of soluble CD4.

To investigate the mechanism of the observed decreases in SIVmac239 Env function, we examined the binding of radiolabeled gp120 to the receptors, CD4 and CCR5. The CCR5 binding experiments were carried out in the presence of soluble CD4. Neither CD4 nor CCR5 binding were affected significantly by the introduced changes (Figure 4), as determined by statistical analysis using GraphPad Prism software where p<0.05 was considered significant. Thus, the changes in the V2 region did not globally disrupt the conformation of monomeric gp120, which is required for binding both receptors.

### Twin-cysteine Changes Cause gp120 Shedding

**Figure 5:**
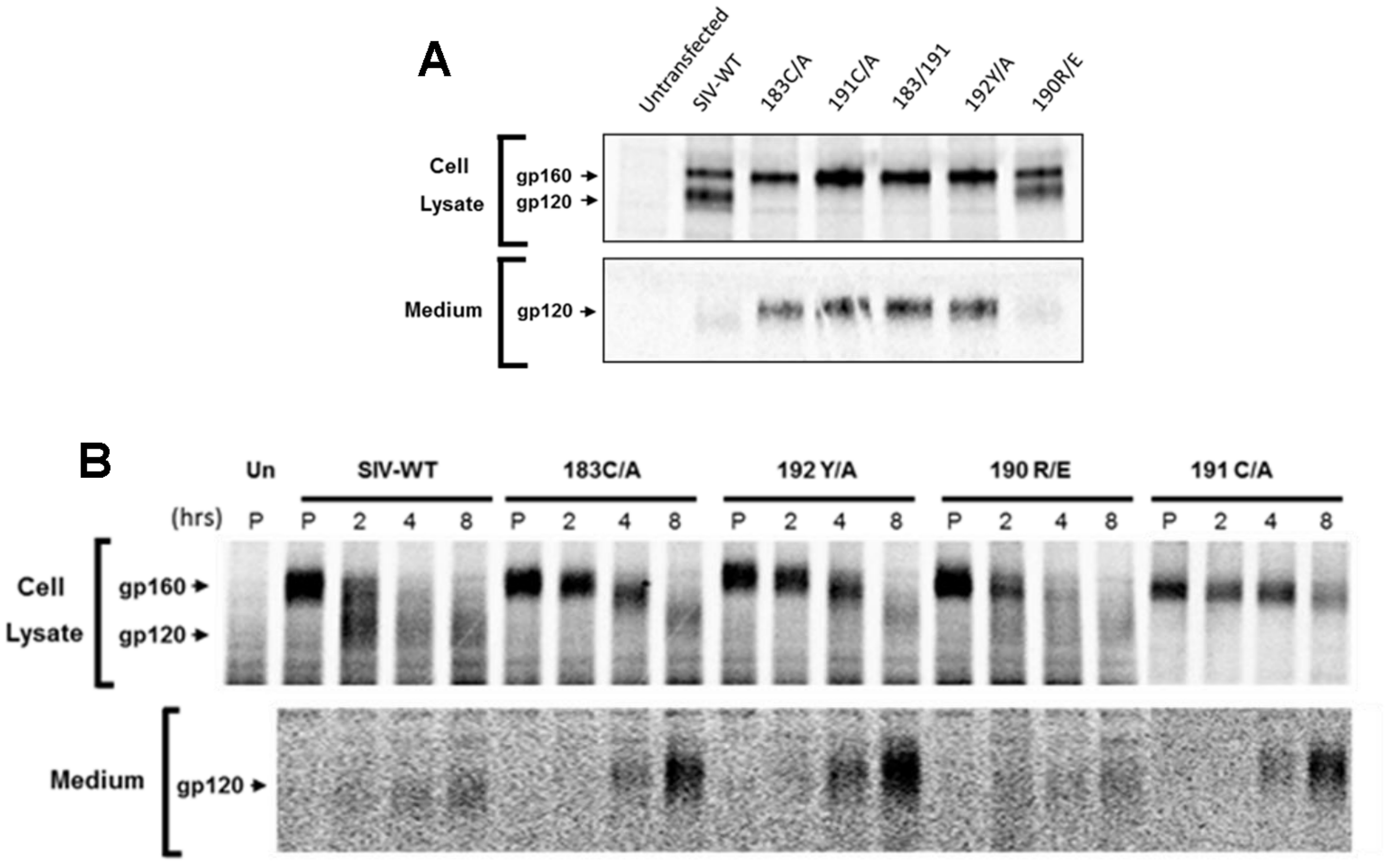
Expression, processing and subunit association of SIVmac239 variants. Cells expressing the SIVmac239 Env variants were labeled with ^35^S-(methioine and cysteine) either continuously overnight (**A**) or in a pulse-chase experiment (**B**). The cells were pulsed in ^35^S-labeling medium for 60 min (P), and then chased for 2, 4 and 8 hours. Un = untransfected cells.

Because of the association of the V2 region with the gp120 TAD, we hypothesized that the twin cysteines may contribute to stabilization of the Env trimer. We examined Env trimer stability by determining the amount of the Env glycoprotein precursor (gp160) and mature gp120 glycoprotein in the cells, as well as shed gp120 in the medium of Env-expressing cells. As shown in Figure 5A, all the SIVmac239 mutants expressed Env well and also processed the gp160 precursor efficiently. The wild-type and 190R/E gp120 glycoproteins were primarily cell-associated, with a relatively small amount of shed gp120 detectable in the medium. By contrast, the single-cysteine mutants 183C/A and 191C/A, the double-cysteine mutant (183C/A+191C/A), and the 192Y/A mutant exhibited significantly higher levels of gp120 in the medium compared to wild-type SIVmac239 Env. The levels of cell-associated gp120 were dramatically reduced for these mutants. Thus, changes in cysteines 183 and 191 or the adjacent conserved tyrosine 192 resulted in a significant decrease in the association of gp120 with the Env trimer.

To confirm the above results, we conducted a pulse-chase experiment on cells expressing the wild-type and mutant SIVmac239 Envs (Figure 5B). A slight delay in the rate of gp160 precursor processing was observed for the twin-cysteine mutants and the 192Y/A mutant, relative to the wild-type SIVmac239 Env. These mutant gp120 glycoproteins were shed into the medium soon after proteolytic processing of the gp160 Env precursor. These results support the observation that changes in the twin cysteines decrease the association of SIVmac239 gp120 with the Env trimer.

## Discussion

Here we show that alteration of the twin-cysteine motif disrupts the stable and non-covalent association of SIVmac239 gp120 with the Env trimer. Presumably as a result of the instability of the mutant Env trimers, these Env mutants exhibit very poor function in cell-cell fusion or virus entry. We suggest that the twin cysteines may form a disulfide-bond to stabilize the Env trimer. This disulfide bond could be formed either within the gp120 subunit or between adjacent subunits. Although there is currently no evidence for inter-protomer disulfide bonds in the SIVmac239 Env trimer, the close association of the V2 regions in the gp120 TAD raises this possibility. Future studies will explore the formation and biological relevance of disulfide bonds between Env protomers. The adjacent tyrosine 192 of the twin-cysteine motif is markedly conserved in SIV, HIV-2 and HIV-1, and change of this residue also interrupts the trimer association. It is indicated that this tyrosine 192 may also play an important role in the Env trimer stabilization.

The twin cysteines in the V2 regions of the gp120 trimer association domain are very conserved in SIV and HIV-2, but are not present in any HIV-1 strains. The majority of SIVcpz strains lack these two cysteine residues, whereas some strains have two cysteine residues in the same vicinity. The twin cysteines appear to have evolved in several SIV lineages, but were minimally retained in SIVcpz and then lost completely in HIV-1. Of interest, during zoonotic transmission from monkeys and establishment in hominoids, PLVs became progressively more dependent on CD4. This increased dependence on CD4 was accompanied by changes in the Phe 43 cavity and inner domain of gp120 [8]–[11]. These changes are predicted to make the gp120 core of HIV-1 less prone to assume the CD4-bound conformation than the SIV gp120 core. The propensity of the gp120 core to adopt the CD4-bound conformation is restrained by the V1/V2 and V3 regions (the TAD) in the unliganded Env trimer [8]–[11]. Given the greater propensity of the SIV gp120 core to assume the CD4-bound state, it is tempting to speculate that the SIVs required more stable TAD association to prevent premature triggering of Env. The ability of the twin-cysteine pair to form a disulfide bond may stabilize TAD inter-protomer interactions and help to maintain the unliganded state of the Env trimer.

Modulation of Env trimer stability may have implications for viral evasion of the host immune response and pathogenicity. The added TAD stability that SIV and HIV-2 gain from structural elements like the twin cysteines may influence the elicitation and efficiency of neutralizing antibodies. Although SIV and HIV-2 elicit a host immune response in the form of neutralizing antibodies, both viruses persist, achieving a balance between virus infection and the host immune reaction. As a result, SIV does not typically cause serious disease in monkey hosts. Similarly, HIV-2 infection in humans is much less likely to progress to full-blown AIDS than HIV-1 infection. As CD4-dependence is associated with greater resistance to neutralization antibodies, HIV-1 may have evolved greater CD4 dependence to escape host immune responses. Poorer host control of HIV-1 is consistent with its pathogenicity. Investigating the functional phenotypes associated with Env diversity in the HIV-1 and HIV-2/SIV lineages should lead to a better understanding of PLV pathogenesis in primate species.
